# Protective effects of medicinal plant-derived metabolites on slow transit constipation via the ENS-ICC-SMC pathway

**DOI:** 10.3389/fphar.2025.1598806

**Published:** 2025-06-11

**Authors:** Zu Gao, Liwen Fu, Wenjun Bai, Junwei Liang

**Affiliations:** ^1^ College of Traditional Chinese Medicine, Shandong University of Traditional Chinese Medicine, Jinan, China; ^2^ Innovative Institute of Chinese Medicine and Pharmacy, Shandong University of Traditional Chinese Medicine, Jinan, China; ^3^ Affiliated Hospital of Shandong University of Traditional Chinese Medicine, Jinan, China

**Keywords:** slow transit constipation, metabolites from botanical drugs, traditional Chinese medicine, pharmacological effects, ENS-ICC-SMC pathway

## Abstract

Slow transit constipation (STC) is a type of functional constipation resulting from a lack of intestinal motility. The primary symptoms are challenging defecation and desiccated feces, which may readily result in perianal, cardiovascular, cerebrovascular, and psychological disorders. The structural and morphological impairment of the enteric nervous system (ENS), along with the dysfunction of interstitial cells of Cajal (ICCs) and smooth muscle cells (SMCs), are the primary contributors to the onset and progression of STC. In recent years, traditional Chinese medicine (TCM) has emerged as an alternative for the therapeutic prevention and treatment of STC. Metabolites obtained from botanical drugs, including quercetin and nobiletin, may ameliorate symptoms of STC, presenting a possible therapeutic approach for STC. This review summarizes metabolites derived from botanical drugs, including flavonoids, terpenoids, steroids, polysaccharides, anthraquinones, and phenylpropanoids, emphasizing their regulatory mechanisms in the treatment of STC via the ENS-ICC-SMC pathway, while also addressing future challenges and developmental directions.

## Graphical Abstract

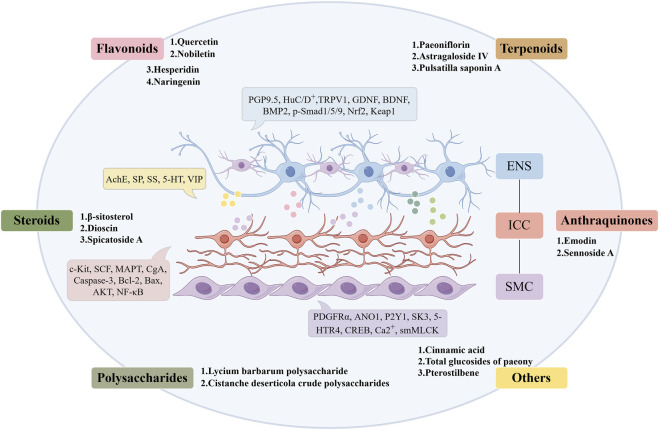

## 1 Introduction

Slow transit constipation (STC) is a refractory form of constipation occurring from numerous non-organic causes of colonic motility problems, leading to delayed passage of intestinal contents. STC mainly presented with difficult defecation, dry stool, abdominal pain and distension (Vlismas et al., 2024). The worldwide prevalence of STC is 14%, whereas among the senior population, it may attain 18% (Black and Ford, 2018; Chu et al., 2014). Laxatives and surgical intervention are prevalent therapies for restoring intestinal motility in patients with STC; however, they are associated with complications including electrolyte imbalance, intestinal mucosal discoloration, postoperative abdominal pain, intestinal obstruction, and recurrence, all of which significantly diminish patients’ quality of life (Ji et al., 2023). Impaired gastrointestinal peristalsis due to intestinal motility disorder is the primary factor contributing to the onset and progression of STC, whereas the enteric nervous system (ENS), interstitial cells of Cajal (ICCs), and smooth muscle cells (SMCs) are essential for sustaining normal gastrointestinal motility (Holland et al., 2025; Zheng et al., 2021; Mazzone et al., 2020).

The ENS is an extensive and intricate network of ganglionic plexuses composed of neurons, nerve fibers, and glial cells situated within the stomach wall (Nguyen et al., 2023; Chalazonitis and Rao, 2018). The ENS can elicit reflex intestinal contractions without central nervous system (CNS) involvement and can autonomously regulate gastrointestinal motility and secretory processes (Benskey et al., 2015). Neurotransmitters released by neurons in the ENS constitute the fundamental basis for the regulation of gastrointestinal function. This includes excitatory neurotransmitters such as acetylcholine (ACh) and Substance P (SP), inhibitory neurotransmitters like vasoactive intestinal peptide (VIP) and nitric oxide (NO), as well as bidirectional neurotransmitters such as 5-hydroxytryptamine (5-HT), all of which collectively influence gastrointestinal motility (Chen et al., 2021; Hansen, 2003). Nonetheless, the ENS does not directly convey signals to the SMCs; rather, there exists an intermediary signal transmission medium, specifically ICCs. Within the gastrointestinal tract, ICCs are situated adjacent to the myenteric nerve plexus and gastrointestinal motor neurons, establishing synaptic connections with nerve terminals. They transfer both excitatory and inhibitory signals to SMCs via gap junctions, resulting in the relaxation and contraction of SMCs (Du et al., 2011). Furthermore, ICCs can generate slow waves and transmit them to SMCs, together governing intestinal contraction and peristalsis (Huizinga et al., 2021). Simultaneously, injury to SMCs, as the ultimate regulators of intestinal motility, will immediately result in diminished intestinal peristalsis and the development of STC (Chen et al., 2014). The ENS-ICC-SMC pathway indicates that neuronal decrease and dysfunction, neurotransmitter imbalance, morphological alterations in ICCs, and injury to SMCs can precipitate the onset of STC. Consequently, identifying alternate techniques and pharmacological agents to enhance STC by modulating the ENS-ICC-SMC pathway is a critical issue that requires resolution.

Prior research indicates that metabolites of botanical drugs can markedly alleviate symptoms of STC by modulating neurotransmitter levels, including serotonin (5-HT) and vasoactive intestinal peptide (VIP), within the compromised ENS, as well as neurotrophic factors such as glial-derived neurotrophic factor (GDNF) and brain-derived neurotrophic factor (BDNF) (Ji et al., 2019; Huang et al., 2023), while also facilitating the repair of damaged ICCs and SMCs (Liu et al., 2024). This review will offer a thorough examination of *in vivo* and *in vitro* tests and outline the research advancements on the metabolites from botanical drugs in enhancing STC via the ENS-ICC-SMC pathway (Table 1).

**TABLE 1:** Metabolites from botanical drugs modulate STC by influencing the ENS-ICC-SMC pathway.

Metabolites	Test subject	Dosage of drugs	The medication time	Control drugs	Mechanism	Site of action	Ref.
Quercetin	Rats	Gavage of aqueous solution (10, 25, 50 mg/kg)	24 h	—	Upregulation: MTL, Gas, AchE, SP, c-Kit, SCF, GDNF Downregulation: SS, TRPV1, NOS.	ICC, Neurotransmitters	Liu and Zhi. (2021)
Nobiletin	Rats	Gavage of aqueous solution (10, 20, 40 mg/kg)	14 days	Mosapride (2.5 mg/kg)	Upregulation: c-kit, SCF Downregulation: 5-HT, VIP, NO, NOS.	ICC, Neurotransmitters	Huang et al. (2023)
Nobiletin	Mouse, Primary ICCs	Gavage of aqueous solution (100, 300 mg/kg)	70 days	Mosapride (2.5 mg/kg)	Upregulation: Ki-67, PCNA, Bcl2 Downregulation: MAPT, TNF-α, IL-1β, IL-6, IFN-γ, P38, JNK, ERK, NF-κB p65, Bax Inhibit MAPK pathway	ICC, Neurotransmitters	Zhou et al. (2024)
Hesperidin	Rats	Gavage of aqueous solution (50,100,200 mg/kg)	7 days	Macrogol 4,000 Powder (0.9 g/kg)	Upregulation: 5-HT, 5-HT4R, PGP9.5, PDGFRα, ANO1, P2Y1, c-kit, SK3	ENS, ICC, SMC, Neurotransmitters	Zhan et al. (2023)
Hesperidin	Rats, Primary smooth muscle cells	Gavage of aqueous solution (50,100,200 mg/kg)	14 days	—	Upregulation: 5-HTR4, Ca^2+^, ADCY3, cAMP, PKA, CREB, p-CREB Activate the cAMP/PKA、p-CREB pathway	SMC	Wu et al. (2020)
Naringenin	Mouse	Gavage of aqueous solution (70, 150, 300 mg/kg)	5 days	—	Upregulation: MTL, Gas, ET, SP, AChE, GDNF, BDNF, c-Kit, SCF Downregulation: TRPV1, NOS.	ENS, ICC, Neurotransmitters	Yin et al. (2018)
Paeoniflorin	Rats	Gavage of aqueous solution (40 mg/kg)	14 days	lactulose oral solution (3.5 mL/kg)	Upregulation: 5-HT, ASIC3, p-ERK/ERK Downregulation: VIP Activate the ASIC3/ERK pathway	Neurotransmitters	Deng et al. (2024)
Paeoniflorin	Rats, RIN-14B cell	Gavage of aqueous solution (10, 20, 40 mg/kg); Medium solution (20, 40, 80 μg/mL)	14 days; 24 h	—	Upregulation: 5-HT, Tph-1, TGR5, TRPA1, PLC-γ1, PIP2 Activate the TGR5/TRPA1 pathway	Neurotransmitters	Zhan et al. (2021)
Astragaloside IV	Mouse, QGP-1 cell	Gavage of aqueous solution (30 mg/kg); Medium solution (50 μm)	5 days; 24 h	—	Upregulation: CD117, CgA, TPH1, Piezo2, p-p38, p-ERK, Caspase-3, Bcl-2 Downregulation: Caspase-3 p12, Bax Inhibit p38 MAPK and ERK pathways	ICC, ENS	Wan et al. (2023)
Astragaloside IV	Mouse, Primary ICCs	Gavage of aqueous solution (10, 30, 90 mg/kg)	5 days	—	Upregulation: c-Kit, p-P65/P65, pAKT/AKT Activate the AKT/NF-κB pathway	ICC	He et al. (2020)
Pulsatilla saponin A	Rats	Gavage of aqueous solution (100, 150, 300 mg/kg)	14 days	—	Upregulation: 5-HT, c-kit, SCF Downregulation: VIP, NO Activate the c-kit/SCF pathway	ICC, Neurotransmitters	Chen et al. (2023)
β-sitosterol	Mouse	Gavage of aqueous solution (200, 500, 750 mg/kg)	7 days	phenolphthalein tablet (200 mg/mL)	Upregulation: ADRA1A, Myl9, smMLCK, 5-HTR4, 5-HT, AchE, c-Kit, SCF Activate the ADRA1A-MLC pathway	ICC, SMC, Neurotransmitters	Liu et al. (2024)
Dioscin	Mouse	Gavage of aqueous solution (60 mg/kg)	7 days	—	Upregulation: BMP2, p-Smad 1/5/9, HuC/D^+^, AchE Downregulation: iNOS, IL-6, TNF-α, VIP Activate the BMP2/p-Smad1/5/9 pathway	ENS, Neurotransmitters	Ren et al. (2022)
Spicatoside A	Mouse	Gavage of aqueous solution (20 mg/kg)	7 days	—	Upregulation: AChE, IP3, C-kit, PGP9.5 Downregulation: Bax, Bcl2, CD34, Gα, p-PKC/PKC, p-PI3K/PI3K, p-MLC/MLC.	ENS, ICC, SMC, Neurotransmitters	Kim et al. (2019)
Lycium barbarum polysaccharide	Rats	Gavage of aqueous solution (40, 80, 120 mg/kg)	14 days	Mosapride (2.5 mg/kg)	Upregulation: SP, C-kit, SCF, Bcl-2 Downregulation: VIP, Bax, Caspase-3	ICC, Neurotransmitters	Liu et al. (2025)
Cistanche deserticola crude polysaccharides	Mouse	Gavage of aqueous solution (100, 200, 400 mg/kg)	7 days	—	Upregulation: SP, SOD, GSH, HuC/D^+^, Nrf2, HO-1, NQO1, GCLC, GCLM Downregulation: VIP, MDA, Keap1 Activate the Nrf2/Keap1 pathway	ENS, Neurotransmitters	Jiang et al. (2024)
Emodin	Mouse	Gavage of aqueous solution (50 mg/kg)	7 days	—	Upregulation: 5-HTR4, GDNF, BDNF, c-Kit, SCF Downregulation: NO, VIPR1, TRPV 1, NOS.	ENS, ICC, Neurotransmitters	Ji et al. (2019)
Sennoside A	Rats, Smooth muscle strips	Gavage of aqueous solution (1 mg/kg)	30 min	—	Upregulation: SP, MOT Downregulation: VIP, CGRP Activate HCN1 channel on ICC membrane	ICC, Neurotransmitters	Li (2019)
Cinnamic acid	Rats	Gavage of aqueous solution (40, 80 mg/kg)	28 days	Prucalopride (0.26 mg/kg)	Upregulation: 5-HT Downregulation: VIP.	Neurotransmitters	Jiang et al. (2023)
Total glucosides of paeony	Rats	Gavage of aqueous solution (0.18 g/kg)	14 days	—	UpregulationSP. Downregulation: NO, NOS, VIP.	ICC, Neurotransmitters	Zhu et al. (2016)
Pterostilbene	Mouse	0.5% carboxymethylcellulose sodium solution by gavage (30, 60 mg/kg)	7 days	—	Upregulation: c-kit, SCF, GSH-Px, p-AKT/AKT, Nrf2, HO-1 Downregulation: caspase-3, MDA Regulates the PI3K/AKT and Nrf2/HO-1 pathways	ICC	Yao et al. (2022)

## 2 Methods of data acquisition

The original articles included in this study were sourced from the PubMed, Web of Science, and CNKI databases. The search terms comprised “slow transit constipation,” “traditional Chinese medicine,” “metabolites from botanical drugs,” “pharmacology,” “enteric nervous system,” “interstitial cells of Cajal,” “smooth muscle cells,” “neurotransmitter,” and their permutations. The search concluded on 1 March 2025, with no prior time constraints imposed. The criteria for inclusion and exclusion were as follows: 1) The pharmacological mechanisms of metabolites from botanical medications in the treatment of STC; 2) The metabolites demonstrate anti-STC actions via the ENS, ICCs, or SMCs; studies unrelated to the ENS-ICC-SMC pathway were omitted. In the screening process, we first examined titles and abstracts to find pertinent research that satisfied the inclusion criteria, followed by an analysis of full-text publications for comprehensive evaluation, culminating in the inclusion of 21 articles.

## 3 Metabolites classification

### 3.1 Flavonoids

Flavonoids constitute a category of metabolites characterized by a fundamental C6-C3-C6 backbone and a core structure of 2-phenylchromogen (Tan et al., 2022). Flavonoids are prevalent in botanical drugs, fruits, and vegetables, exhibiting diverse pharmacological actions, including anti-inflammatory (Maleki et al., 2019) and antioxidative stress properties (Huang et al., 2020). Quercetin is one of the metabolites of Ginkgo folium (*Ginkgo biloba* L.) and Scutellariae radix (*Scutellaria baicalensis* Georgi). Researchers developed a rat model of constipation using loperamide to assess the therapeutic efficacy of quercetin. The findings indicated that quercetin enhanced intestinal transport rate, elevated serum motilin (MTL), gastrin (Gas), acetylcholine esterase (AchE), and SP levels, while reducing somatostatin (SS) expression in constipated rats. Simultaneously, quercetin can upregulate the mRNA of c-kit proto-oncogene protein (c-Kit), stem cell factor (SCF), and GDNF in the intestinal tissue of constipated rats, while downregulating the expression of transient receptor potential vanilloid 1 (TRPV1) and nitric oxide synthase (NOS) (Liu and Zhi, 2021). Nobiletin, a flavonoid derived from the pericarp of Citri reticulatae (*Citrus reticulata* Blanco), exhibits antifungal and anti-inflammatory properties. Nobeletin enhances the amplitude of slow wave frequency in colon electromyography in STC mice, diminishes the levels of 5-HT, NO, and NOS in colon tissue, and elevates the protein expression of c-kit and SCF, which are markers for ICCs in the colon (Huang et al., 2023). Nobiletin diminished the expression of microtubule-associated protein-tau (MAPT) in colon tissue and decreased the levels of tumor necrosis factor-α (TNF-α), interleukin-1β (IL-1β), interleukin-6 (IL-6), interferon-γ (IFN-γ), and mitogen-activated protein kinase (MAPK) pathway-related proteins in the serum of STC mice. Nobiletin can inhibit the apoptosis of ICCs *in vitro*, which is related to the inhibition of MAPT expression and the activation of MAPK pathway in ICCs of STC mice (Zhou et al., 2024). Hesperidin and naringenin, metabolites obtained from Aurantii fructus and Aurantii fructus immaturus (*Citrus aurantium* L.), exhibit notable therapeutic effects on STC. Hesperidin can restore colonic motility and the morphology of damaged colonic tissue in STC rats, potentially by activating the 5-HT signaling pathway, upregulating the expression of anoctamin 1 (ANO1), c-kit, platelet-derived growth factor receptor α (PDGFRα), P2Y purinoceptor 1 (P2Y1), and KCa2.3 (SK3), thereby restoring the phenotype and function of ICCs and PDGFRα^+^ cells, and enhancing SIP syncytia function (Zhan et al., 2023). Moreover, hesperidin can rehabilitate the gastrointestinal transmission function in STC rats, augment the quantity of colonic SMCs and neurons, and stimulate the proliferation of SMCs *in vitro*, potentially linked to the upregulation of the 5-Hydroxytryptamine receptor 4 (5-HTR4) and intracellular calcium ion concentration. Furthermore, the upregulation of cyclic adenosine monophosphate (cAMP)/protein kinase A (PKA) pathway and p-cAMP-response element binding protein (CREB) pathway-related protein expression is related (Wu et al., 2020). The research indicated that naringenin may elevate serum levels of MTL, Gas, endothelin (ET), SP, AChE, and VIP in STC mice, while downregulating TRPV1 and NOS in colonic tissue, and upregulating the protein and mRNA levels of GDNF and BDNF. Naringenin simultaneously upregulated the expression levels of the ICCs marker proteins c-Kit and SCF (Yin et al., 2018).

### 3.2 Terpenoids

Terpenoids refer to all isoprene polymers and their derivatives, which exhibit various biological activities, including antioxidative stress and antiviral properties (Lin et al., 2018; Gupta et al., 2017). Paeoniflorin is a monoterpene glycoside metabolite extracted from botanical drugs. An animal study revealed that paeoniflorin can mitigate constipation symptoms and colonic pathological damage in STC rats, enhance abnormal visceral sensitivity, potentially by elevating serum 5-HT and decreasing VIP levels, up-regulating acid-sensitive ion channel 3 (ASIC3) and phosphorylated extracellular signal-regulated kinase (p-ERK)/ERK protein and mRNA levels, and activating the ASIC3/ERK pathway (Deng et al., 2024). Paeoniflorin can markedly enhance the necrosis of colonic mucosal epithelial cells and the infiltration of inflammatory cells in STC rats, while also facilitating the release of 5-HT from enterochromaffin cells, potentially through the activation of the G Protein-Coupled BA Receptor 1 (TGR5)/transient receptor potential ankyrin 1 (TRPA1) pathway (Zhan et al., 2021). Astragaloside IV is one of the main metabolites of Astragali radix (*Astragalus membranaceus* (Fisch.) Bge. var. *mongholicus* (Bge.) Hsiao), exhibiting many actions including the scavenging of oxygen free radicals, anti-inflammatory properties, and enhancement of cardiovascular function. A study demonstrated that astragaloside IV enhances the intestinal propulsion rate in STC mice, improves ICCs, and mitigates the loss of enterochromaffin cells by elevating the expression of CD117 and chromogranin A (CgA) proteins in colon and cecum tissues. The safeguarding of enterochromaffin cells by astragaloside IV may be facilitated through the activation of p38 and ERK pathways (Wan et al., 2023). In a separate study utilizing 16S rRNA microbial analysis, astragaloside IV augmented the quantity of ICCs in the colons of STC mice and elevated the amount of *Lactobacillus* reuteri and butyrate synthesis. Butyrate can facilitate defecation, enhance intestinal motility, and stimulate ICCs proliferation by modulating the protein kinase B (AKT)/nuclear factor kappa-B (NF-κB) signaling pathway (He et al., 2020). Pulsatilla saponin A is one of the metabolites from Pulsatillae radix (*Pulsatilla chinensis* (Bge) Regel). It can markedly augment the quantity of fecal granules, ameliorate colon pathological damage, elevate serum 5-HT levels, diminish VIP and NO levels, and upregulate c-kit and SCF protein expression in the colon tissue of STC rats. This suggests that pulsatilla saponin A may alleviate constipation symptoms in STC rats via modulating the c-kit/SCF pathway and subsequently influencing the levels of enteric neurotransmitters (Chen et al., 2023).

### 3.3 Steroids

Steroids are a category of chemicals characterized by a cyclopentane pyranophenylene structure, exhibiting diverse biological functions including anti-inflammatory and antioxidant effects (Shi et al., 2019; Abo-Zaid et al., 2023). β-sitosterol is one of the metabolites of Gastrodiae rhizoma (*Gastrodia elata* Bl.). A study demonstrated that β-sitosterol could enhance the frequency and rate of bowel movements in STC mice, while also upregulating the mRNA and protein expression of adrenoceptor alpha 1 A (ADRA1A) and myosin regulatory light chain 9 (Myl9) in the colon, thereby facilitating intestinal peristalsis and activating the ADRA1A/myosin light chain (MLC) signaling pathway. Simultaneously, β-sitosterol can modulate serum 5-HT, AchE, and colon 5-HT4, SCF, c-Kit, and smMLCK mRNA expression to alleviate constipation symptoms (Liu et al., 2024). Dioscin is one of the metabolites of Dioscoreae rhizoma (*Dioscorea opposita* Thunb.). It can regenerate HuC/D^+^ neurons in STC mice via activating the bone morphogenetic protein 2 (BMP2)/p-Smad1/5/9 signaling pathway, elevate AchE levels in colon tissue, and diminish levels of inducible nitric oxide synthase (iNOS), IL-6, and TNF-α. The microenvironment of the ENS can be enhanced, the myenteric nerve plexus can be restructured, and gastrointestinal hormone levels can be modulated to alleviate constipation (Ren et al., 2022). Spicatoside A is a steroid metabolite extracted from Liriope spicata Lour. The management of STC may involve the upregulation of AChE levels and the downregulation of p-protein kinase C (PKC)/PKC and p-phosphoinositide 3-kinase (PI3K)/PI3K in colonic mucosa. Subsequent research has demonstrated that Spicatoside A can enhance the production of c-Kit and protein gene product 9.5 (PGP9.5) while reducing the levels of p-MLC/MLC in colon tissue, consequently preventing neuronal degeneration and enhancing the functionality of ICCs and SMCs (Kim et al., 2019).

### 3.4 Polysaccharides

Polysaccharides are natural high polymers often generated by the condensation of several monosaccharide molecules with the concomitant loss of water. They possess a diverse array of biological activities and applications (Yu et al., 2018). Lycium barbarum polysaccharide, mainly obtained from Lycii fructus (*Lycium barbarum* L.), were observed to elevate serum secretion of SP, reduce secretion of VIP, enhance expression of C-kit, SCF, and B-cell lymphoma-2 (Bcl-2), while inhibiting the expression of Bcl-2 associated X protein (Bax) and Caspase-3 in the colon tissues of STC rats. This indicates that LBP may mitigate the symptoms of STC and improve gastrointestinal peristalsis by modulating gastrointestinal hormone levels, fostering proliferation, and suppressing the apoptosis of ICCs (Liu et al., 2025). Cistanche deserticola crude polysaccharide is one of the metabolites derived from the fleshy stems of the dried scaly leaves of Cistanches herba (*Cistanche deserticola* Y. C. Ma). One study showed that it had neuroprotective effects on STC mice. It may markedly alleviate constipation symptoms in STC mice, elevate serum SP levels, diminish VIP levels, block the degeneration of myenteric neurons, and decrease the number of nNOS^+^ neurons. It can diminish mitochondrial oxidative stress and dysfunction of colonic myenteric neurons by elevating superoxide dismutase (SOD) and glutathione (GSH) levels while reducing malondialdehyde (MDA) content. This regulation may be related to the upregulation of nuclear factor erythroid 2 - related factor 2 (Nrf2) and downregulation of kelch - like ECH - associated protein 1 (Keap1) protein levels, the upregulation of heme oxygenase - 1 (HO-1), NAD (P) H quinone oxidoreductase 1 (NQO1), Glutamate - cysteine ligase catalytic subunit (GCLC) and Glutamate - cysteine ligase modifier subunit (GCLM) mRNA levels, and the activation of Nrf2/Keap1 pathway (Jiang et al., 2024).

### 3.5 Anthraquinones

Anthraquinones with the parent nucleus structure of anthraquinone have a variety of pharmacological effects such as laxative, antibacterial, anti-tumor and anti-oxidation (Lu et al., 2024). Emodin is one of the metabolites of Rhei radix et rhizoma (*Rheum palmatum* L.). A study demonstrated that emodin can decrease serum NO levels and the expression of VIP receptor 1 (VIPR1), TRPV1, and NOS in the colonic tissue of STC mice, while enhancing the expression of 5-HTR4, GDNF, BDNF, c-Kit, and SCF, thus mitigating loperamide hydrochloride-induced STC by rectifying ENS dysfunctions (Ji et al., 2019). Sennoside A is the main metabolite of Sennae folium (*Cassia angustifolia* Vah). A study revealed that sennoside A can augment the frequency and amplitude of slow waves in ICCs within the gastrointestinal tract of STC mice by activating the HCN1 channel on the ICCs membrane. Sennoside A can elevate the concentrations of SP and motilin (MOT) in the plasma and colonic tissue of rats with STC, while concurrently decreasing levels of VIP and calcitonin gene-related peptide (CGRP), ultimately enhancing gastrointestinal peristalsis and demonstrating a beneficial therapeutic impact on STC (Li, 2019).

### 3.6 Others

Besides the aforementioned metabolites, more classes exist that potentially influence STC therapeutically by influencing essential signaling pathways. Cinnamic acid, a natural phenylpropanoid metabolite, enhances the frequency and pace of defecation in STC rats and repairs the compromised intestinal mucosa. This may pertain to the upregulation of serum 5-HT and downregulation of VIP levels, modulating the α and β variety of the intestinal microbiome, and enhancing the quantity of intestinal bacteria (Jiang et al., 2023). Total glucosides of paeony, derived from Paeoniae radix alba (*Paeonia lactiflora* Pall.), can enhance fecal volume, moisture content, and intestinal transit rate in the STC rat model. The proposed mechanism of action involves the downregulation of serum NO, NOS, and VIP, alongside the upregulation of SP content, and the enhancement of c-kit and SCF protein levels in colon tissue to ameliorate dysfunctional ICCs (Zhu et al., 2016). Pterostilbene is the antifungal metabolite of Draconis sanguis. It can reduce the expression of caspase-3 protein and increase the expression of c-kit and SCF protein in the colon tissue of STC rats, indicating that pterostilbene can improve the symptoms of STC constipation by reducing ICCs apoptosis and activating SCF/C-Kit pathway. Further study found that pterostilbene could reduce serum MDA and increase GSH-Px content in STC mice, and increase the protein expression of p-AKT/AKT, Nrf2 and HO-1 in colon tissue. This suggests that pterostilbeni treatment of STC is achieved by inhibiting oxidative stress through PI3K/AKT signaling mediated by its downstream Nrf2/HO-1 signaling to reduce the apoptosis of ICCs (Yao et al., 2022).

## 4 Conclusion and prospects

**FIGURE 1:**
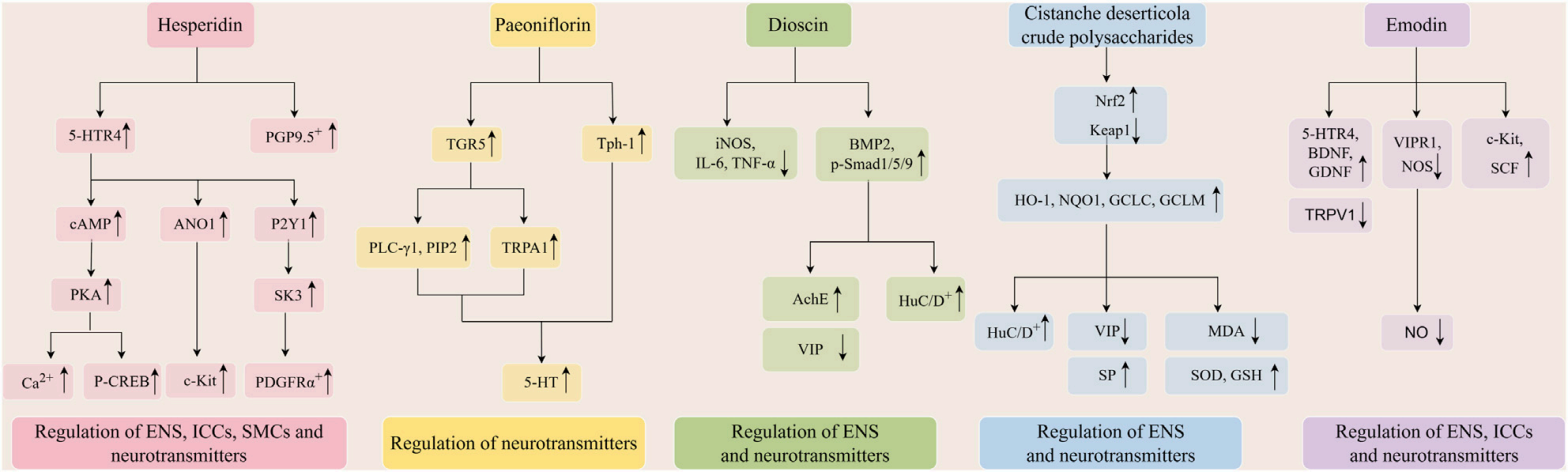
The pathways of main metabolites from botanical drugs in the treatment of STC.

In this study, 17 metabolites derived from botanical drugs were summarized, including quercetin, nobiletin, hesperidin, naringenin, paeoniflorin, astragaloside IV, pulsatilla saponin A, β-sitosterol, dioscin, spicatoside A, lycium barbarum polysaccharide, cistanche deserticola crude polysaccharides, emodin, sennoside A, cinnamic acid, total glucosides of paeony, pterostilbene. They contribute to the enhancement of STC by modulating neurotransmitter release, mending the compromised ENS, and reinstating the functions of ICCs and SMCs (Figure 1). Despite the potential of these metabolites in the prevention and treatment of STC, current research presents certain limitations: (1) The majority of research on the metabolites relies on animal studies and *in vitro* cellular models, with a deficiency of pertinent clinical trials to substantiate the precise efficacy of these metabolites. (2) Pan-Assay Interference Compounds (PAINS) are increasingly acknowledged by individuals. Phenols, quinones, flavonoids, steroids, and triterpenes are prevalent substances in botanical drugs, as well as in common PAINS. They have multi-target and multi-pathway therapeutic features and demonstrate effective binding to the protein active site; however, their lack of selectivity poses obstacles for drug screening. (3) Currently, pharmacological research on the metabolites of botanical drugs lacks a systematic and complete framework. Numerous research exhibit persistent and fundamental issues, necessitating comprehensive and systematic investigations across holistic, organ, cellular, and molecular dimensions. (4) Despite the potential effects of these metabolites in animal or cellular models, their toxicity and side effects remain ambiguous and require further assessment through additional animal studies and clinical trials. (5) Many metabolites exhibit inadequacies, including malabsorption and accelerated metabolism; thus, the issue of drug administration must be addressed by integrating the disease’s location or utilizing novel materials. In conclusion, metabolites from botanical drugs possess significant potential as supplementary and alternative therapies for the treatment of STC. Future research should concentrate on observing clinical efficacy, thoroughly investigating pharmacological mechanisms, and assessing drug safety and administration routes.
